# Supplementary material for: SPARK regulates AGC kinases central to the Toxoplasma gondii asexual cycle
Source: bioRxiv. 2024 May 2:2023.10.30.564746. Originally published 2023 Oct 30. Preprint. [Version 2] doi: 10.1101/2023.10.30.564746 (PMC10634940; doi:10.1101/2023.10.30.564746)
Supplement: Supplement 6 [file NIHPP2023.10.30.564746v2-supplement-6.pdf]

bioRxiv preprint doi: https://doi.org/10.1101/2023.10.30.564746; this version posted May 2, 2024. The copyright holder for this preprint (which was not certified by peer review) is the author/funder, who has granted bioRxiv a license to display the preprint in perpetuity. It is made available under aCC-BY 4.0 International license.

SOURCE DATA

Figure 2—source data 1. This file contains source data that was used to generate the blot in Figure 2B. V5, LICOR.

Figure 2—source data 2. This file contains source data that was used to generate the blot in Figure 2B. TUB1, LICOR.

Figure 2—source data 3. This file contains source data that was used to generate the blot in Figure 2C. V5, LICOR.

Figure 2—source data 4. This file contains source data that was used to generate the blot in Figure 2C. CDPK1, LICOR.

Figure 2—figure supplement 1 source data 1. This file contains source data that was used to generate the blot in Figure 2—figure supplement 1B. V5, LICOR.

Figure 2—figure supplement 1 source data 2. This file contains source data that was used to generate the blot in Figure 2—figure supplement 1B. ALD1, LICOR.

Figure 6—source data 1. This file contains source data that was used to generate the blot in Figure 6D. V5, LICOR.

Figure 6—source data 2. This file contains source data that was used to generate the blot in Figure 6D. ALD1, LICOR.

Figure 7—source data 1. This file contains source data that was used to generate the blot in Figure 7D. mNG, LICOR.

Figure 7—source data 2. This file contains source data that was used to generate the blot in Figure 7D. V5, LICOR.

Figure 7—source data 3. This file contains source data that was used to generate the blot in Figure 7D. ALD1, LICOR.

bioRxiv preprint doi: https://doi.org/10.1101/2023.10.30.564746; this version posted May 2, 2024. The copyright holder for this preprint (which was not certified by peer review) is the author/funder, who has granted bioRxiv a license to display the preprint in perpetuity. It is made available under aCC-BY 4.0 International license.

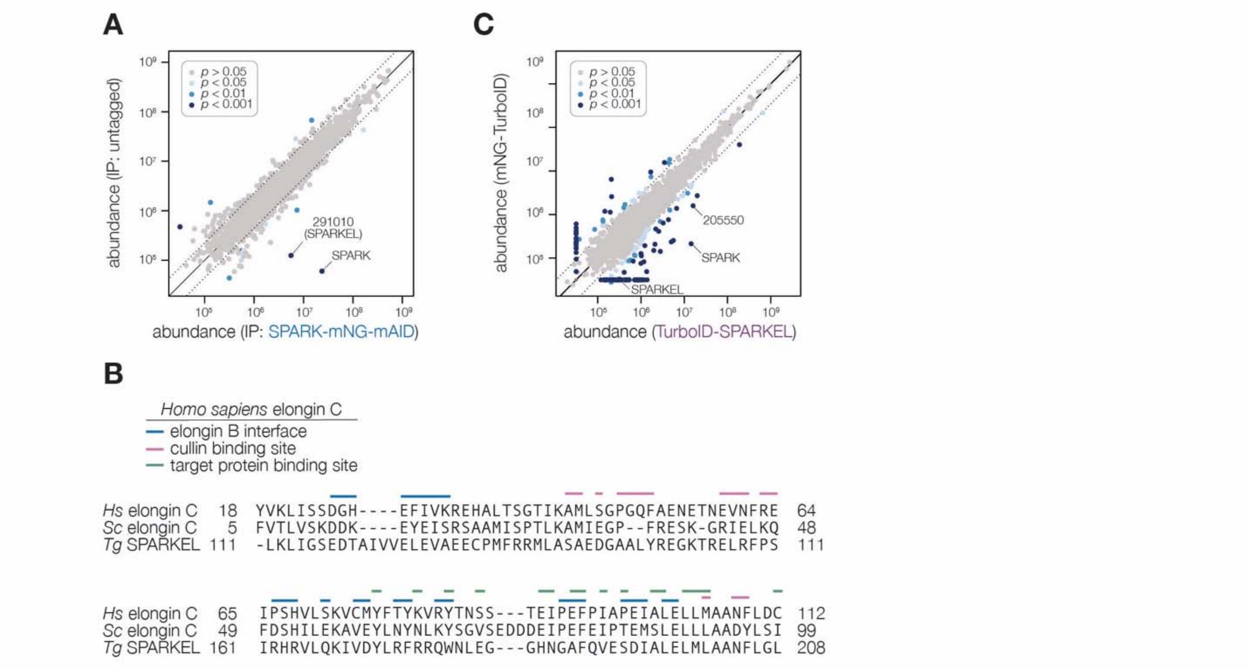

Figure 1—figure supplement 1. Additional data supporting the interaction between SPARK and SPARKEL. (A) Protein abundances from immunopurified SPARK-mNG-mAID (Smith et al., 2022) lysates or an untagged control strain. Dotted lines correspond to one modified z-score. Only proteins quantified by greater than one peptide are shown. Proteins identified in only one IP were assigned a pseudo-abundance of $10^{4.5}$. (B) Alignment of the SKP1/BTB/POZ domains of Homo sapiens elongin C, S. cerevisiae elongin C, and SPARKEL. The elongin B interface, cullin binding sites, and target protein binding sites based on the Homo sapiens annotation are shown. **(C)** Protein abundances following biotinylation and streptavidin enrichment of samples derived from parasites expressing mNG- or TurboID-SPARKEL fusion constructs. A pseudocount of $10^{4.5}$ was assigned to proteins identified in only one sample. Point colors correspond to significance thresholds. Dotted lines correspond to one median absolute deviation.

bioRxiv preprint doi: https://doi.org/10.1101/2023.10.30.564746; this version posted May 2, 2024. The copyright holder for this preprint (which was not certified by peer review) is the author/funder, who has granted bioRxiv a license to display the preprint in perpetuity. It is made available under aCC-BY 4.0 International license.

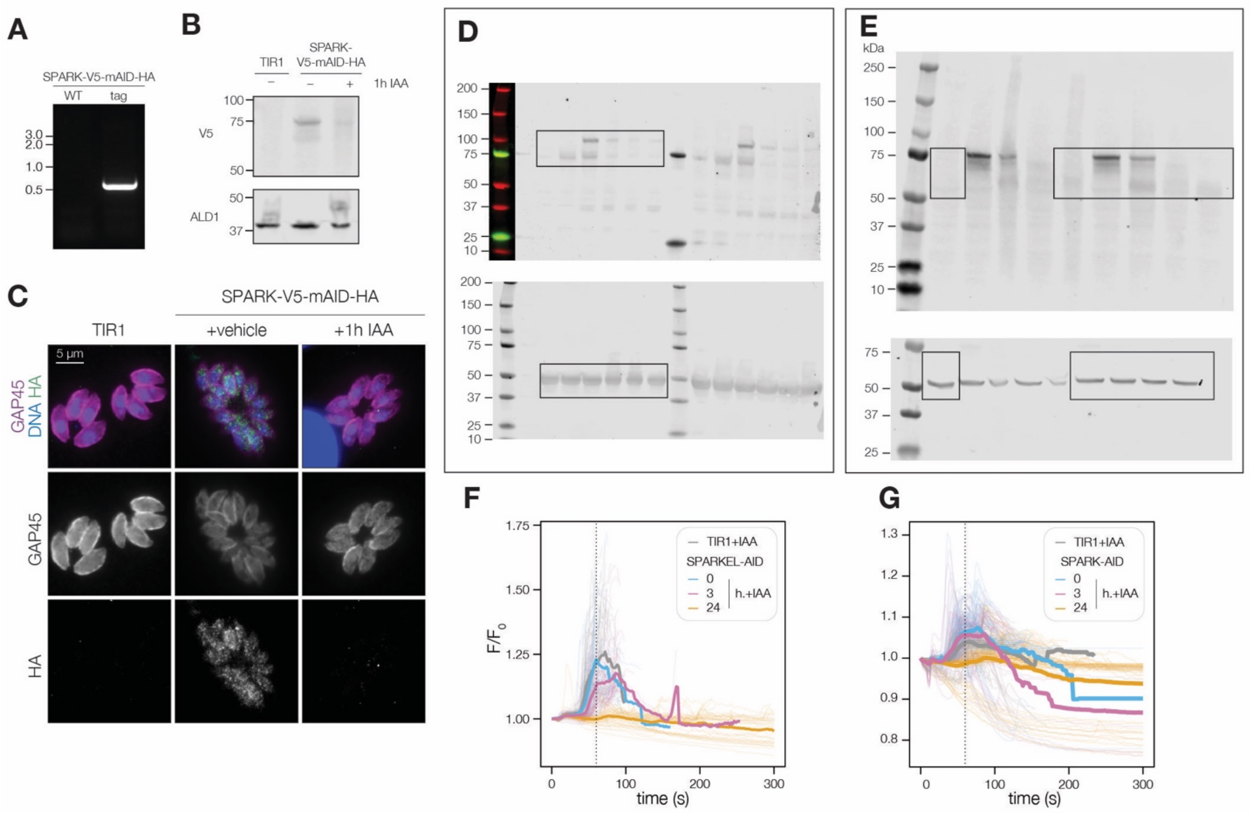

Figure 2—figure supplement 1. (A) Primers specific to the 3′ terminus of SPARK and the V5-mAID-HA tagging payload amplified a product in the SPARK-V5-mAID-HA strain but not the untagged parental strain. **(B)** Confirmation of SPARK-V5-mAID-HA depletion via immunoblot using the V5 epitope. ALD1 was used as a loading control. **(C)** SPARK-V5-mAID-HA depletion was visualized in formaldehyde-fixed intracellular parasites using the HA epitope and GAP45 staining as a parasite marker. DNA was visualized with Hoechst. HA signal intensity was normalized relative to the TIR1 parental strain. **(D)** Uncropped immunoblots corresponding to **Figure 2B**. **(E)** Uncropped immunoblots corresponding to **Figure 2C**. **(F, G)** Normalized GCaMP6f fluorescence of individual SPARK-AID and SPARKEL-AID vacuoles, respectively, after zaprinast treatment and prior to egress (transparent lines) for the indicated period of IAA treatment. The solid line represents the mean normalized fluorescence of all vacuoles across *n* = 3 biological replicates.

bioRxiv preprint doi: https://doi.org/10.1101/2023.10.30.564746; this version posted May 2, 2024. The copyright holder for this preprint (which was not certified by peer review) is the author/funder, who has granted bioRxiv a license to display the preprint in perpetuity. It is made available under aCC-BY 4.0 International license.

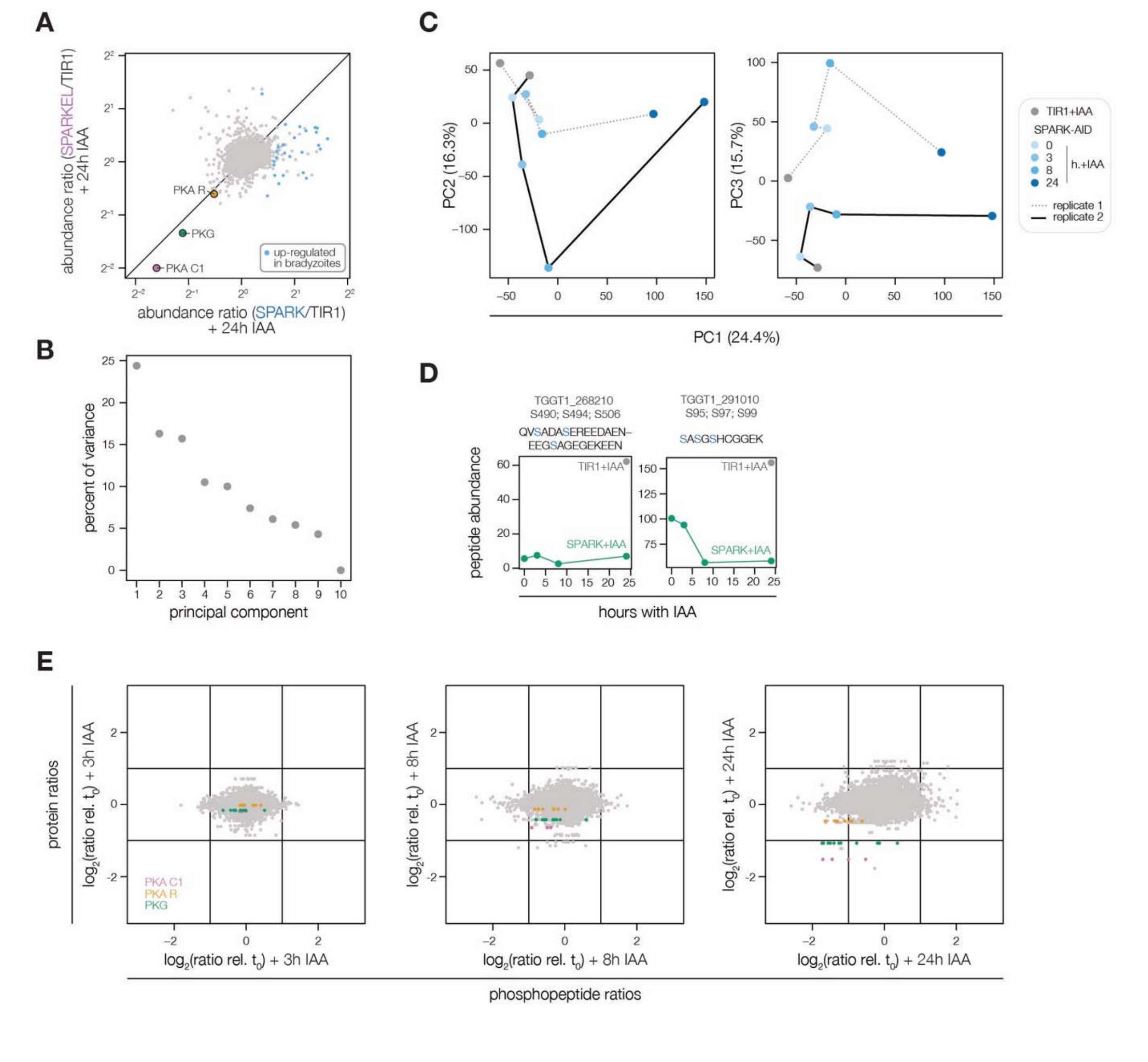

Figure 3—figure supplement 1. Extended analysis of the SPARK-AID depletion phosphoproteome. **(A)** Protein abundance ratios of SPARK- or SPARKEL-AID parasites treated with IAA for 24 h relative to the untreated samples. Enriched proteins identified as up-regulated in alkaline-induced bradyzoites (Waldman et al., 2020) are shown in blue. The points corresponding to PKA C1, PKA R, and PKG are highlighted in pink, orange, and green, respectively. **(B, C)** Principal component analysis of the SPARK-AID depletion phosphoproteome. Plots show the three components accounting for the greatest proportion of the variance **(D)** Abundances of SPARK and SPARKEL peptides detected by mass spectrometry. (E) Protein or phosphopeptide abundance ratios from SPARK-AID parasites treated with IAA for the indicated number of h relative to untreated samples. Values corresponding to PKA C1, PKA R, and PKG are color-coded.

bioRxiv preprint doi: https://doi.org/10.1101/2023.10.30.564746; this version posted May 2, 2024. The copyright holder for this preprint (which was not certified by peer review) is the author/funder, who has granted bioRxiv a license to display the preprint in perpetuity. It is made available under aCC-BY 4.0 International license.

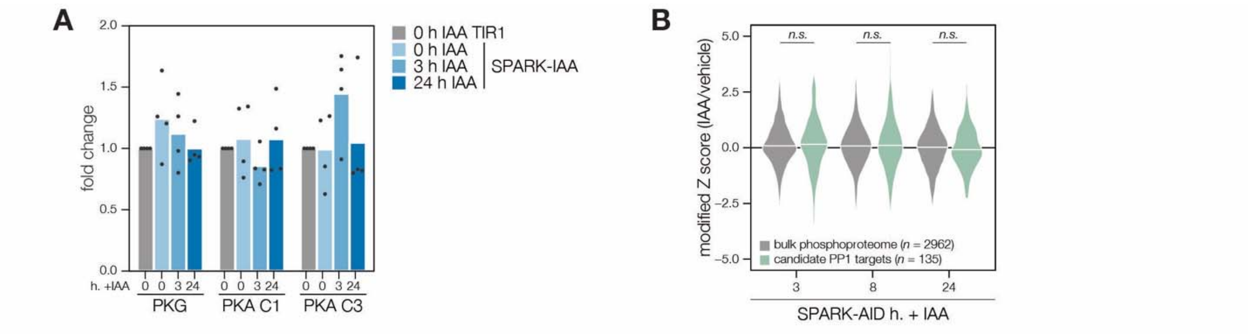

Figure 4—figure supplement 1. Transcriptional and phosphoproteomic effects of SPARK depletion. (A) Transcript abundances for PKG, PKA C1, and PKA C3 were compared by qRT-PCR in TIR1 and SPARK-AID, treated with IAA for 0, 3, or 24 h. Expression levels were normalized to the parental TIR1 strain across n = 4 biological replicates. (B) Overlap between the SPARK and PP1 phosphoproteomes. Violin plots displaying the distribution of phosphopeptide abundance values following SPARK depletion. The distributions of candidate PP1 targets, as defined in the text and methods, are shown in green. The distributions and p-values (KS test) were derived from the overlapping subset of phosphopeptides identified in each dataset. PP1 proteome data was obtained from (Herneisen et al., 2022).

bioRxiv preprint doi: https://doi.org/10.1101/2023.10.30.564746; this version posted May 2, 2024. The copyright holder for this preprint (which was not certified by peer review) is the author/funder, who has granted bioRxiv a license to display the preprint in perpetuity. It is made available under aCC-BY 4.0 International license.

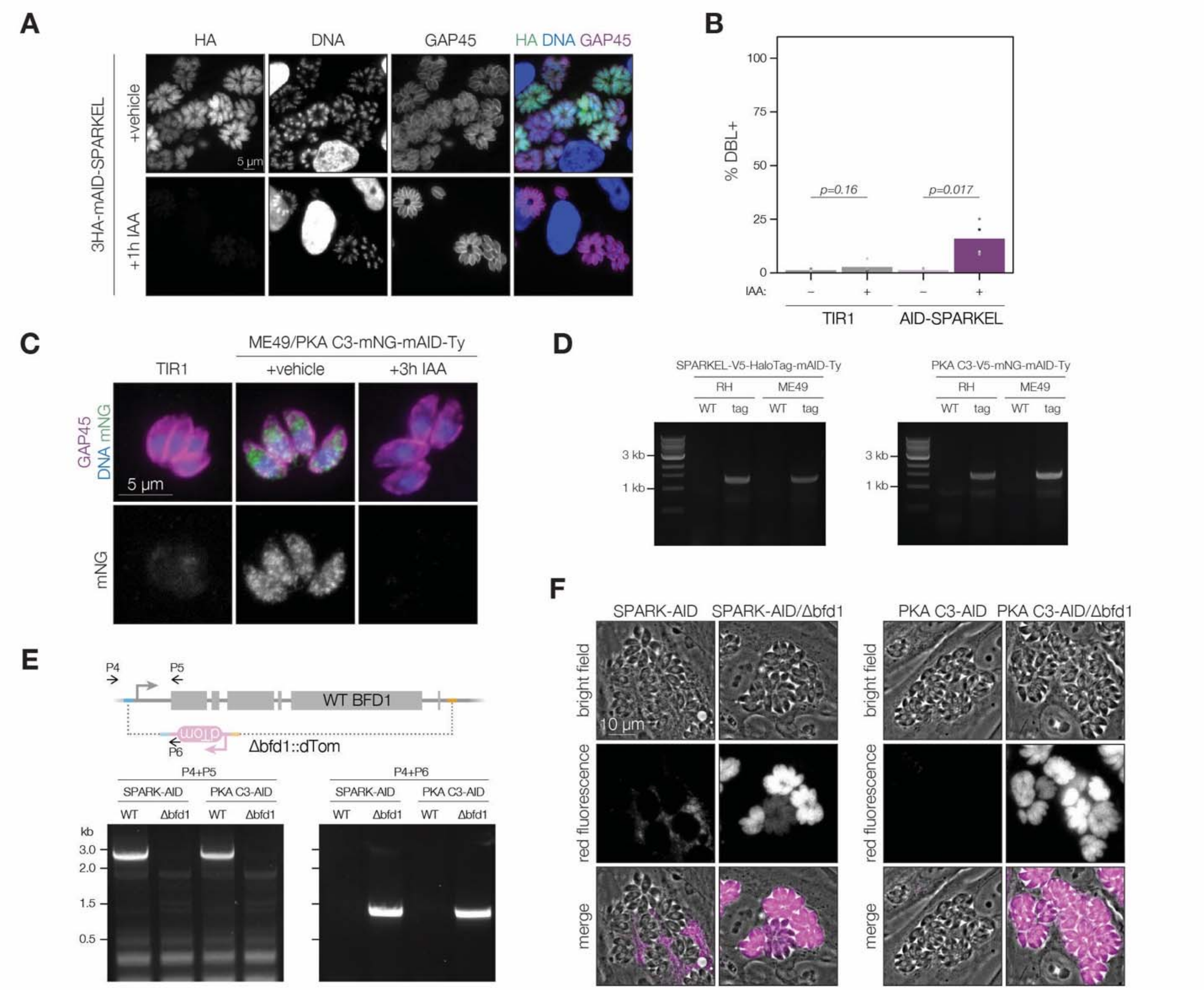

Figure 6—figure supplement 1. (A) Fixed, intracellular RH/3HA-mAID-SPARKEL parasites visualized by immunofluorescence microscopy using the HA epitope after 1h of vehicle or IAA treatment. The HA signal was normalized to the vehicle-treated sample. **(B)** Quantification of the number of DBL+ vacuoles expressed as a percentage of the total stained vacuoles following 48h of AID-SPARKEL knockdown. Two-sided t-test. **(C)** Fixed, intracellular ME49/PKA C3-mNG-AID parasites visualized by immunofluorescence microscopy using the mNG epitope after 3h of vehicle or IAA treatment. The mNG signal was normalized to the vehicle-treated sample. **(D)** Amplification of the SPARKEL-AID and PKA C3-AID genomic loci using tag-specific primers to confirm correct integration of the tagging payload. The integration was confirmed with Sanger sequencing between the 3′ gene junction and CDPK3 3′UTR from the tag (Smith et al., 2022). **(E)** The strategy used to knock out and replace BFD1 with a dTomato cassette containing homology to sequenced flanking the BFD1 locus (Waldman et al., 2020). Amplification of sequences specific to the intact locus or dTom knockout for the indicated strains are shown below the schematic. Oligonucleotide sequences are listed in **Supplementary Table 5**. **(F)** Live microscopy images of intact or Δbfd1::dTom parasites showing red fluorescence arising from the knockout cassette.

bioRxiv preprint doi: https://doi.org/10.1101/2023.10.30.564746; this version posted May 2, 2024. The copyright holder for this preprint (which was not certified by peer review) is the author/funder, who has granted bioRxiv a license to display the preprint in perpetuity. It is made available under aCC-BY 4.0 International license.

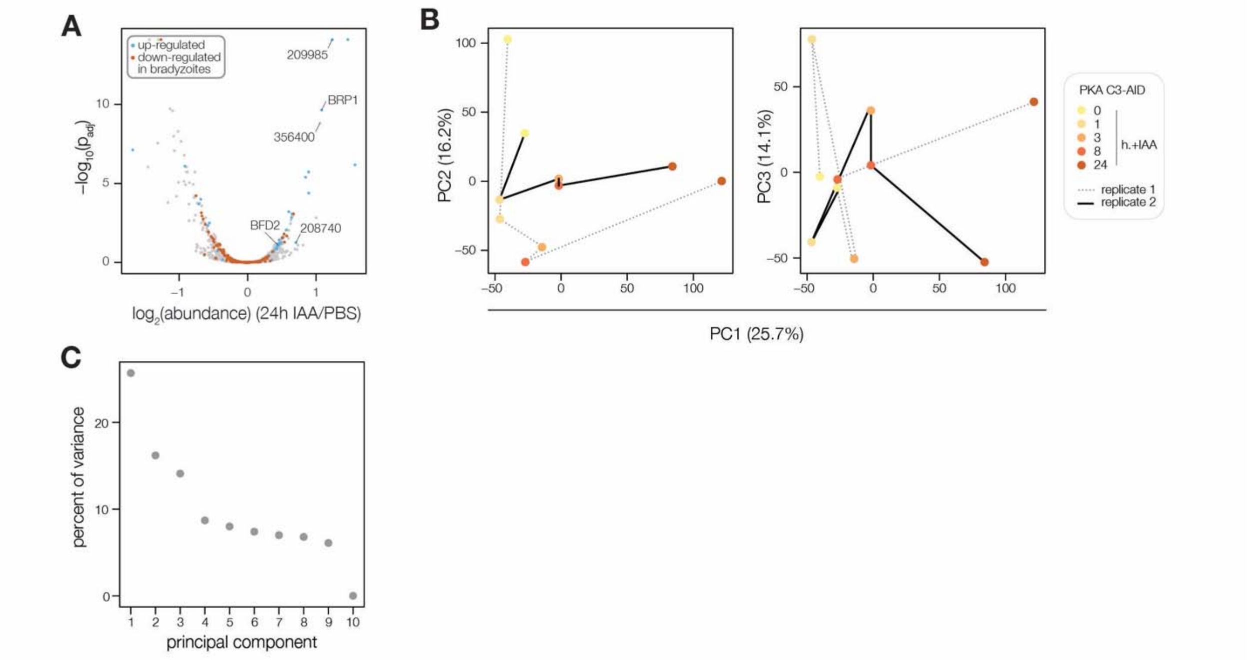

Figure 7—figure supplement 1. Extended analysis of the PKA C3 depletion proteome. (A) Volcano plot displaying the protein abundance ratios of SPARK-AID parasites treated with IAA or vehicle for 24 h and adjusted p-values. Proteins identified as up- or down-regulated in parasites overexpressing the driver of differentiation (BFD1) (Waldman et al., 2020) are shown in blue and vermilion, respectively. **(B, C)** Principal component analysis of the PKA C3-AID depletion phosphoproteome. Plots show the three components accounting for the greatest proportion of the variance.
